# Prevalence and Incidence of Amyotrophic Lateral Sclerosis in Japan

**DOI:** 10.2188/jea.JE20140059

**Published:** 2014-11-05

**Authors:** Yuriko Doi, Naoki Atsuta, Gen Sobue, Mitsuya Morita, Imaharu Nakano

**Affiliations:** 1 Area on Epidemiological Research, National Institute of Public Health, Saitama, Japan; 1 国立保健医療科学院疫学調査研究分野; 2 Department of Neurology, Nagoya University Graduate School of Medicine, Nagoya, Japan; 2 名古屋大学大学院医学系研究科神経内科学; 3 Division of Neurology, Department of Medicine, Jichi Medical University School of Medicine, Tochigi, Japan; 3 自治医科大学内科学講座神経内科学部門; 4 Tokyo Metropolitan Neurological Hospital, Tokyo, Japan; 4 東京都立神経病院

**Keywords:** ALS, amyotrophic lateral sclerosis, epidemiology, incidence, Japan

## Abstract

**Background:**

Previous studies have reported a high incidence of amyotrophic lateral sclerosis (ALS) in endemic foci in the Kii Peninsula, Japan. However, little is known about the ALS frequency in the whole country. Furthermore, the presence of ethnic variation in the incidence of ALS remains unknown.

**Methods:**

We conducted a nationwide survey of ALS frequency in 2013 to estimate its annual prevalence and incidence. ALS was diagnosed based on the El Escorial Criteria. The study period was the 2009 fiscal year, from April 2009 to March 2010. To compare the incidence of ALS among prefectures, standardized incidence ratios (SIRs) and 95% confidence intervals (CIs) were calculated under the assumption of Poisson distribution.

**Results:**

The annual crude prevalence and incidence rates per 100 000 people per year were 9.9 (95% CI 9.7–10.1) and 2.2 (95% CI 2.1–2.3), respectively. The age group with the highest prevalence as well as incidence was 70–79 years, and the male-female ratio was approximately 1.5. The annual incidence rate adjusted for age and sex using the 2000 U.S. standard population was 2.3 (95% CI 2.2–2.4) per 100 000 people. Some prefectures had significantly high SIRs: Okinawa, Nara and Wakayama in the Kii Peninsula, and Niigata for males; Kumamoto for females.

**Conclusions:**

This is the first report on the annual prevalence and incidence of ALS in the representative population of Japan. We identified some prefectures with a high incidence of ALS. However, the incidence of ALS in the Japanese population was much lower than in the Caucasian populations of Europe and North America.

## Abstract (ja)

**背景：:**

先行研究では、紀伊半島に筋萎縮性側索硬化症（ALS）の高い罹患の集積があったとの報告などがあるが、日本全体でのALSの頻度についてはよくわかっていない。さらに、ALSの罹患に人種間の差があるかどうかについても、よくわかっていない。

**方法：:**

我々は、ALSの年間有病率と罹患率を推定するために、ALSの頻度に関する全国調査を2013年に実施した（回収率100％）。本研究におけるALSとは、対象年度（2009年度（2009年4月1日～2010年3月31日））にEl Escorial の診断基準にもとづき診断されたものを言う。なお、ALSの罹患率を比較するにあたり、ポアソン分布をするという前提のもとで、標準化罹患率と95％信頼区間を算出した。

**結果：:**

2009年度の粗有病率（/10万人/年）と粗罹患率（/10万人/年）は、それぞれ9.9（9.7—10.1）と2.2（2.1—2.3）であった。年齢別にみると、粗有病率および粗罹患率ともに、70歳代で最も高く、性別をみると、男性の方が女性より約1.5倍ほど高かった。47都道府県別に標準化罹患比をみてみると、男性では沖縄、奈良、和歌山、新潟、女性では熊本で高かった。人種間の比較を行うために、米国の2000年基準人口を用いて標準化罹患率（/10万人/年）を求めたところ、2.3（2.2—2.4）という結果であった。

**結論：:**

本研究は、日本人における年間有病率および罹患率に関する、初めての報告と言える。国内においては、一部の都道府県に高い罹患率が見られ、ALSの罹患に地域差が示唆された。海外との比較においては、欧米の白色人種に比べると、その罹患率は極めて低いことが示唆された。

## INTRODUCTION

Amyotrophic lateral sclerosis (ALS) is a progressive and fatal neurodegenerative disease characterized by the selective loss of upper and lower motor neurons. A total of 5%–10% of cases of ALS are familial, with the remainder believed to be sporadic.^1^ The incidence of ALS is uniform across Caucasian populations,^2^ but the presence of ethnic variation remains unknown.^3^

The Western Pacific form of ALS, termed ALS and parkinsonism-dementia complex (ALS/PDC), was identified in the 1950s in three distinct geographic isolates: Guam, Western New Guinea, and the Kii Peninsula of Japan.^4^ The high prevalence and incidence of ALS/PDC has been reported in the Kii Peninsula (eg a prevalence of 47.7 per 100 000 people and an incidence of 9.54 per 100 000 person-years in the Kozagawa focus area of Wakayama prefecture).^5^ However, little is known about the ALS frequency across Japan as a whole.

In this study, we estimate the prevalence and incidence of ALS in Japan. Furthermore, we compare the incidence between Japan and other countries, and examine geographic variation in the incidence of ALS among prefectures within the country.

## METHODS

### Specified disease treatment research program in Japan

Medical care is ensured for all people in Japan under the universal healthcare system, in which a 30% patient copayment is required for insurance-covered medical care. Some rare and intractable diseases, termed “specified diseases,” remain difficult to treat, develop chronically, and constitute a great financial burden for patients and their family because of the high costs associated with long-term care and medicine. The specified disease treatment research program, which started in 1972, subsidizes the patient copayment for insured patients suffering from designated intractable diseases. A patient with ALS can receive that financial support, independent of disease severity.

### Case ascertainment of ALS

When a patient is diagnosed with ALS by their doctor, they can apply to their prefectural government for assistance through the specified disease treatment research program, using the initial clinical application form for a new patient or the renewal form for an existing patient. Specialists in neurology serving on the committee on designated intractable diseases in each prefecture review the clinical application form, which is usually completed by a neurologist, based on the El Escorial Criteria (EEC).^6^^,^^7^ As of 2009, there were 8555 neurologists in Japan.^8^

The criteria for ALS in this program are as follows^9^:

• adult onset, with a steady, progressive course;
• the presence of clinical or electrophysiological evidence of lower motor neuron (LMN) degeneration in at least two topographical anatomic regions (brainstem, cervical, thoracic or lumbosacral region), together with clinical evidence of upper motor neuron (UMN) degeneration in at least one region; and
• the absence of electrophysiological and pathological evidence of other disease processes that might explain the signs of LMN and/or UMN degeneration and neuroimaging evidence of other disease processes that might explain the observed clinical and electrophysiological signs.

According to the criteria, patients with definite, probable, or possible ALS are included in this program, based on the revised EEC.^7^^,^^9^

### Nationwide mail survey on amyotrophic lateral sclerosis

We sent a questionnaire on ALS to all 47 prefectural offices on February 11, 2013, requesting the following information:

• the number of patients with certified ALS who received financial aid for the treatment of a designated intractable disease in the 2009 fiscal year, classified by the categories (i) new or existing patients, (ii) sex, and (iii) age at which they were certified as a patient with ALS in the specified disease treatment research program (Note: patient’s age at the time of diagnosis, application, and certification during the fiscal year is the same in most cases); and
• the total number of neurology specialists and the composition of the committee on designated intractable diseases.

The study period was the 2009 fiscal year, from April 2009 to March 2010, the latest fiscal year before occurrence of the Great East Japan Earthquake on March 11, 2011. We chose to examine events prior the earthquake because of the huge impact that it had on the population structure and quantity and quality of medical institutions and personnel in the Tohoku district, the consequences of which persist to date.

This study was approved by the Institutional Review Board of the National Institute of Public Health, Japan (NIPH-TRN#12009).

### Statistical analysis

The annual prevalence rate was calculated from the observed number of all patients with ALS certified as eligible for financial aid for the treatment of a designated intractable disease during the 2009 fiscal year, divided by the population insured by national insurance. It was obtained by subtracting the population in receiving public assistance on July 31, 2009, from the total population on October 1, 2009. Overall, the insured population comprised approximately 99% of the total population. To calculate the annual incidence rate, the numerator was the observed number of patients with ALS newly certified as eligible for financial aid for the treatment of a designated intractable disease during the 2009 fiscal year. The rates were also calculated separately for age and sex.

To compare the incidence of ALS among different ethnic groups,^3^ the rates were age- and sex-adjusted to the 2000 U.S. standard population using the 50- to 79-years age band by the direct method. We calculated 95% confidence intervals (CIs) under the assumption of Poisson distribution. In addition, geographic variation within the country was examined by calculating standardized incidence ratios (SIRs) and 95% CIs for all 47 prefectures using the indirect method under the assumption of Poisson distribution.^10^^,^^11^ SIR with a 95% CI that did not include 1.0 was considered statistically significant.

## RESULTS

All 47 prefectural offices returned their responses by June 2013. A special effort was made to achieve a response rate of 100% and confirm the data provided. Of 614 physicians comprising the committees on designated intractable diseases, the number of neurology specialists was 100, with at least 1 neurology specialist in the committee for each of 46 prefectures (unknown for Saga prefecture).

Table 1 shows the number of patients with ALS certified as eligible for financial aid for the treatment of a designated intractable disease during the 2009 fiscal year. Of a total of 10 237, including 6 patients younger than 20 years old, 2264 were patients with newly certified ALS.

**Table 1.** Number of patients with ALS certified as eligible for financial aid for the treatment of a designated intractable disease in Japan from April 2009 to March 2010

Age^a^	All patients			New patients		
	
(years)	Both	Male	Female	Both	Male	Female
0–4	0	0	0	0	0	0
5–9	1	1	0	0	0	0
10–14	2	0	2	0	0	0
15–19	3	1	2	1	1	0
20–24	10	8	2	2	2	0
25–29	19	14	5	2	1	1
30–34	50	23	27	8	4	4
35–39	95	58	37	13	7	6
40–44	226	120	106	41	17	24
45–49	319	180	139	55	26	29
50–54	558	326	232	99	54	45
55–59	1065	663	402	223	143	80
60–64	1590	955	635	337	217	120
65–69	1796	1103	693	394	234	160
70–74	1948	1111	837	450	269	181
75–59	1408	752	656	360	194	166
80–84	843	407	436	204	96	108
85+	304	120	184	75	37	38
Total	10 237	5842	4395	2264	1302	962

Regrouped						
20+	10 231	5840	4391	2263	1301	962

^a^Age at which a patient was certified as eligible for financial aid for the treatment of a designated intractable disease from April 2009 to March 2010 (Note: Patient’s age at the time of diagnosis, application and certification during the fiscal year is the same in most cases).

Table 2 shows the annual prevalence and incidence rates of patients with ALS aged 20 years or older. The annual crude prevalence was 9.9 (95% CI 9.7–10.1) per 100 000 people, with the highest age- and sex-specific rate of 27.1 (95% CI 26.2–28.0) per 100 000 people evident in the 70- to 79-years age group. The annual crude incidence was 2.2 (95% CI 2.1–2.3) per 100 000 people, with the highest age- and sex-specific rate of 6.5 (95% CI 6.1–7.0) per 100 000 people evident in the 70- to 79-years age group. Greater numbers of males were evident for both prevalence and incidence. To enhance the comparability of incidence rates among people of different ethnicities, incidence rates were standardized for the 50- to 79-years age band using the 2000 U.S. standard population (Table 2). The rate for both sexes combined was 2.3 (95% CI 2.2–2.4) per 100 000 people.

**Table 2.** Annual prevalence and incidence rates of patients with ALS, aged 20 years or older, certified as eligible for financial aid for the treatment of a designated intractable disease in Japan from April 2009 to March 2010

	Both				Male				Female				M:F ratio
		
Age^a^ (years)	Patients	Population^b^	Rate^c^	95% CI	Patients	Population^b^	Rate^c^	95% CI	Patients	Population^b^	Rate^c^	95% CI	

Prevalence													
20–29	29	14 371 936	0.2	0.1–0.3	22	7 366 821	0.3	0.2–0.5	7	7 005 115	0.1	0.0–0.0	3.0
30–39	145	18 193 826	0.8	0.7–0.9	81	9 241 101	0.9	0.7–1.1	64	8 952 725	0.7	0.6–0.9	1.2
40–49	545	16 253 995	3.4	3.1–3.6	300	8 187 477	3.7	3.3–4.1	245	8 066 518	3.0	2.7–3.4	1.2
50–59	1623	16 631 377	9.8	9.3–10.2	989	8 235 051	12.0	11.3–12.8	634	8 396 326	7.6	7.0–8.2	1.6
60–69	3386	17 419 338	19.4	18.8–20.1	2058	8 393 464	24.5	23.5–25.6	1328	9 025 874	14.7	13.9–15.5	1.7
70–79	3356	12 393 683	27.1	26.2–28.0	1863	5 576 087	33.4	31.9–35.0	1493	6 816 596	21.9	20.8–23.0	1.5
80+	1147	7 736 961	14.8	14.0–15.7	527	2 627 371	20.1	18.4–21.8	620	5 109 590	12.1	11.2–13.1	1.7

Crude	10 231	103 001 116	9.9	9.7–10.1	5840	49 627 372	11.8	11.5–12.1	4391	53 372 744	8.2	8.0–8.5	1.4
Incidence^d^													
20–29	4	14 371 936	0.0	0.0–0.1	3	14 371 936	0.0	0.0–0.1	1	7 005 115	0.0	0.0–0.0	2.9
30–39	21	18 193 826	0.1	0.1–0.2	11	18 193 826	0.1	0.1–0.2	10	8 952 725	0.1	0.1–0.2	1.1
40–49	96	16 253 995	0.6	0.5–0.7	43	16 253 995	0.5	0.4–0.7	53	8 066 518	0.7	0.5–0.9	0.8
50–59	322	16 631 377	1.9	1.7–2.2	197	16 631 377	2.4	2.1–2.8	125	8 396 326	1.5	1.2–1.8	1.6
60–69	731	17 419 338	4.2	3.9–4.5	451	17 419 338	5.4	4.9–5.9	280	9 025 874	3.1	2.7–3.5	1.7
70–79	810	12 393 683	6.5	6.1–7.0	463	12 393 683	8.3	7.6–9.1	347	6 816 596	5.1	4.6–5.7	1.6
80+	279	7 736 961	3.6	3.2–4.1	133	7 736 961	5.1	4.2–6.0	146	5 109 590	2.9	2.4–3.4	1.8

Crude	2263	103 001 116	2.2	2.1–2.3	1301	103 001 116	2.6	2.5–2.8	962	53 372 744	1.8	1.7–1.9	1.5
Age-adjusted^e^			2.3	2.2–2.4			4.7	4.4–5.0			1.7	1.6–1.9	2.8

^a^Age at which a patient was certified as eligible for financial aid for the treatment of a designated intractable disease from April 2009 to March 2010 (Note: Patient’s age at the time of diagnosis, application and certification during the fiscal year is the same in most cases).

^b^The population receiving public assistance on July 31, 2009, is subtracted from the total population on October 1, 2009.

^c^per 100 000 people per year.

^d^The numerator was the number of patients with ALS newly certified as eligible for financial aid for the treatment of a designated intractable disease during the study period.

^e^Age-adjusted incidence was standardized to the 2000 U.S. standard population using the 50- to 79-years age band for ethnic comparison.

Table 3 shows the observed and expected numbers of patients with newly certified ALS, and SIRs with 95% CIs for each prefecture. Among males, SIRs were significantly high in Okinawa, Nara, Wakayama, and Niigata, and significantly low in Tokyo and Osaka. Among females, SIRs were significantly high in Kumamoto and significantly low in Miyazaki.

**Table 3.** Observed and expected numbers of patients with ALS newly certified and the standardized incidence ratio by prefecture in Japan from April 2009 to March 2010

Prefecture	Male (*n* = 1301)			Female (*n* = 962)		
	
	O	E	SIR (95% CI)	O	E	SIR (95% CI)
Hokkaido	67	57.3	1.17 (0.91–1.48)	43	44.4	0.97 (0.70–1.30)
Aomori	16	14.3	1.12 (0.64–1.82)	14	11.8	1.19 (0.65–1.99)
Iwate	16	14.9	1.07 (0.61–1.74)	14	11.8	1.19 (0.65–1.99)
Miyagi	18	23.2	0.77 (0.46–1.22)	11	17.5	0.63 (0.31–1.13)
Akita	12	12.8	0.94 (0.48–1.64)	13	10.4	1.25 (0.66–2.13)
Yamagata	13	13.4	0.97 (0.52–1.66)	14	10.3	1.36 (0.74–2.28)
Fukushima	26	22.0	1.18 (0.77–1.73)	9	16.6	0.54 (0.25–1.03)
Ibaraki	41	31.0	1.32 (0.95–1.79)	21	21.7	0.97 (0.60–1.48)
Tochigi	16	20.7	0.77 (0.44–1.26)	9	14.7	0.61 (0.28–1.16)
Gunma	22	21.3	1.03 (0.65–1.56)	10	15.3	0.65 (0.31–1.20)
Saitama	70	71.9	0.97 (0.76–1.23)	46	48.3	0.95 (0.70–1.27)
Chiba	61	63.1	0.97 (0.74–1.24)	47	43.4	1.08 (0.80–1.44)
Tokyo	97	122.5	0.79 (0.64–0.97)	78	88.4	0.88 (0.70–1.10)
Kanagawa	87	86.1	1.01 (0.81–1.25)	70	59.9	1.17 (0.91–1.48)
Niigata	39	26.5	1.47 (1.04–2.01)	25	20.1	1.24 (0.80–1.83)
Toyama	13	12.1	1.07 (0.57–1.83)	12	9.4	1.28 (0.66–2.24)
Ishikawa	13	12.1	1.08 (0.57–1.84)	10	9.3	1.08 (0.52–1.99)
Fukui	10	8.7	1.16 (0.55–2.12)	4	6.6	0.61 (0.17–1.56)
Yamanashi	11	9.2	1.20 (0.60–2.14)	4	6.8	0.59 (0.16–1.51)
Nagano	24	24.1	0.99 (0.64–1.48)	20	17.8	1.12 (0.68–1.73)
Gifu	32	22.4	1.43 (0.98–2.02)	18	16.3	1.11 (0.66–1.75)
Shizuoka	39	40.5	0.96 (0.68–1.31)	30	29.3	1.03 (0.69–1.47)
Aichi	76	71.3	1.07 (0.84–1.33)	45	49.7	0.91 (0.66–1.21)
Mie	22	19.9	1.10 (0.69–1.67)	8	14.6	0.55 (0.24–1.08)
Shiga	19	13.4	1.42 (0.86–2.22)	8	9.7	0.83 (0.36–1.63)
Kyoto	19	26.4	0.72 (0.43–1.12)	14	19.9	0.70 (0.39–1.18)
Osaka	62	85.7	0.72 (0.55–0.93)	70	63.5	1.10 (0.86–1.39)
Hyogo	43	56.7	0.76 (0.55–1.02)	47	42.4	1.11 (0.81–1.47)
Nara	29	14.8	1.96 (1.31–2.81)	9	10.9	0.82 (0.38–1.56)
Wakayama	20	11.2	1.79 (1.10–2.77)	9	8.7	1.03 (0.47–1.96)
Tottori	7	6.4	1.09 (0.44–2.25)	3	5.1	0.59 (0.12–1.75)
Shimane	14	8.4	1.66 (0.91–2.78)	7	6.6	1.05 (0.42–2.17)
Okayama	24	20.8	1.15 (0.74–1.72)	14	15.7	0.89 (0.49–1.49)
Hiroshima	20	29.7	0.67 (0.41–1.04)	18	22.4	0.80 (0.48–1.27)
Yamaguchi	16	16.5	0.97 (0.55–1.57)	15	13.1	1.14 (0.64–1.88)
Tokushima	11	8.8	1.25 (0.62–2.24)	4	6.8	0.59 (0.16–1.50)
Kagawa	6	11.0	0.54 (0.20–1.19)	11	8.3	1.32 (0.66–2.36)
Ehime	10	15.7	0.64 (0.31–1.17)	15	12.4	1.21 (0.68–2.00)
Kochi	8	8.6	0.93 (0.40–1.84)	7	6.9	1.01 (0.41–2.09)
Fukuoka	43	47.5	0.90 (0.65–1.22)	46	37.9	1.21 (0.89–1.62)
Saga	13	8.7	1.50 (0.80–2.56)	11	7.0	1.57 (0.78–2.81)
Nagasaki	10	15.1	0.66 (0.31–1.22)	11	12.2	0.90 (0.45–1.61)
Kumamoto	21	19.2	1.09 (0.68–1.67)	24	15.3	1.57 (1.01–2.33)
Oita	11	13.1	0.84 (0.42–1.50)	14	10.3	1.35 (0.74–2.27)
Miyazaki	15	12.3	1.22 (0.68–2.02)	3	9.6	0.31 (0.06–0.92)
Kagoshima	16	18.3	0.87 (0.50–1.42)	16	14.6	1.09 (0.63–1.78)
Okinawa	23	11.4	2.02 (1.28–3.04)	11	8.3	1.32 (0.66–2.36)

## DISCUSSION

This is the first report on the prevalence and incidence of ALS in a representative population of Japan. The annual crude prevalence and incidence rates per 100 000 people per year were 9.9 (95% CI 9.7–10.1) and 2.2 (95% CI 2.1–2.3), respectively. The highest prevalence as well as incidence was evident in the 70- to 79-years age group, and the male-female ratio was approximately 1.5.

According to the *Report on Public Health Administration and Services 2009*,^12^ the number of patients with ALS-awarded certificates of financial aid for the treatment of a designated intractable disease at the end of the 2009 fiscal year was 8492, which is 1745 fewer than the total of 10 237 identified in our study. The certificates must be returned to the governor of the patient’s prefecture if they die. This difference almost corresponds to the number of deaths due to ALS between January 1 and December 31, 2009, recorded as 1797 in the country’s vital statistics. We therefore believe that the published value in the report, given as 6.7 per 100 000 people, underestimates the annual crude prevalence of ALS, which has a high fatality rate.

How ethnicity influences an individual’s risk of developing ALS is of great concern. A systematic review on the effect of ethnic variation on the incidence of ALS, using an incidence rate standardized to the 2000 U.S. standard population, provides some etiologic clues.^3^ The age- and sex-adjusted incidence rate of our study, 2.3 per 100 000 people per year, was comparable to the rate of 2.0 per 100 000 person-years reported in Hokkaido, Japan.^3^^,^^13^ These incidence rates are much lower than those reported in recent prospective studies: 6.4 in Ireland, 6.2 in Scotland, 5.5 in Italy (Northern), 5.3 in the U.S. (Washington), and 5.0 in Italy (Puglia); as well as in retrospective studies: 6.6 in the U.S. (Minnesota), 6.0 in Sweden, 5.6 in Canada, 5.3 in Norway, 4.6 in Libya, and 4.2 in Denmark.^3^ In general, a low incidence is believed to be caused by the low occurrence of a disease, poor access to medical care, short life expectancy, and inter-disease competition.^3^^,^^14^ These latter three conditions are unlikely to account for the low incidence of ALS in Japan, which provides ALS patients with subsidized public medicine and whose population has the longest life expectancy in the world. Therefore, if the difference is genuine, genetic or lifestyle factors that protect against ALS may confer a low risk of ALS in the Japanese population.

In addition, we found variation in the incidence of ALS among prefectures in Japan. Although the reason for that variation was unclear, the combined data of familial and sporadic ALS used in this study might reflect in part the geographic variation of genetic and/or environmental factors contributing to the occurrence of ALS. Among males, SIRs were high in Okinawa, in Nara and Wakayama on the Kii Peninsula, and in Niigata. Okinawa is where hereditary motor and sensory neuropathy with proximal dominancy (HMSN-P) was first reported in 1997.^15^ The clinical features of HMSN-P include adult-onset ALS accompanied by mild sensory disturbance, progressing to bedridden incapacity.^16^ Although HMSN-P is inherited as an autosomal dominant characteristic, some patients with HMSN-P may have been included in this study. According to recent reports, a high incidence of ALS persisted in males in Wakayama prefecture,^17^^,^^18^ which is consistent with the findings of our study. Although it remains unclear why the SIR is high in Nara, one possible explanation is the immigration of residents with a family history of ALS from two major endemic foci in Wakayama and Mie prefectures to Nara prefecture in the Kii Peninsula. Future research is required to confirm the above hypothesis by comparing patients’ place of birth with place of ALS diagnosis, using information obtained from the clinical application form.

Similarly, the reasons underlying the high SIR in Niigata among males are unclear. A previous mortality study reported that deaths among males due to ALS most often occurred throughout Niigata, Gunma, Nagano, and Fukushima prefectures.^19^ Considering these findings together, we may speculate that some unidentified foci with a high incidence of ALS exist in this region. Conversely, Tokyo and Osaka presented a slightly lower SIR in males, but the statistical significance was modest. For female patients, SIR was high in Kumamoto but low in Miyazaki; the statistical significance was modest. However, the reason for this discrepancy among prefectures was unclear.

The limitations of our present study should be taken into account. One is the case ascertainment of ALS. As described in the methods, however, this is not so serious, considering the following points: (1) ALS was usually diagnosed by neurologists; (2) the number of neurologists was large^8^; (3) neurologists have generally followed the clinical guidelines based on the EEC since the recommendation of its use by the research committee on ALS of Japan^9^; and (4) the agreement between judgments by the doctor who diagnosed and filled out the clinical application form and neurology specialists who reviewed it at the committee on designated intractable diseases was quite high.^20^ The other is potential regional disparity affecting geographic variation in the incidence of ALS (eg number of neurologists and access to specialized medical care). Given the following point, however, the degree of regional disparity is likely to be small: prefectures with higher or lower concentrations of neurology clinics/departments^21^ were not consistent with those in which the SIRs of ALS were high or low in our present study. Basically, all ALS patients have been guaranteed free medical access thanks to the provision of financial aid from universal medical insurance since 1961, countermeasures against intractable diseases including ALS since 1972, and nursing-care insurance since 2000.

In conclusion, we report here the annual prevalence and incidence of ALS in a representative population of Japan. We identified some prefectures with a high incidence of ALS. However, the incidence of ALS in the Japanese population was much lower than in Caucasian populations of Europe and North America.

## ONLINE ONLY MATERIAL

Abstract in Japanese.
