# Supplementary material for: Prevalence and Incidence of Amyotrophic Lateral Sclerosis in Japan
Source: J Epidemiol. 2014 Nov 5;24(6):494–9. doi: 10.2188/jea.JE20140059 (PMC4213224; doi:10.2188/jea.JE20140059)
Supplement: Abstract in Japanese. [file je-24-494-s001.pdf]

タイトル

日本における筋萎縮性側索硬化症の有病率と罹患率

著者

土井由利子[1]、熱田直樹[2]、祖父江元[2]、森田光哉[3]、中野今治[4]

[1]国立保健医療科学院疫学調査研究分野、[2]名古屋大学大学院医学系研究科神経内科学、[3]自治医科大学内科学講座神経内科学部門、[4]東京都立神経病院

背景 : 先行研究では、紀伊半島に筋萎縮性側索硬化症（ALS）の高い罹患の集積があったとの報告などがあるが、日本全体での ALS の頻度についてはよくわかっていない。さらに、ALS の罹患に人種間の差があるかどうかについても、よくわかっていない。

方法 : 我々は、ALS の年間有病率と罹患率を推定するために、ALS の頻度に関する全国調査を 2013 年に実施した（回収率 100%）。本研究における ALS とは、対象年度（2009 年度（2009 年 4 月 1 日～2010 年 3 月 31 日））に El Escorial の診断基準にもとづき診断されたものを言う。なお、ALS の罹患率を比較するにあたり、ポアソン分布をするという前提のもとで、標準化罹患率と 95%信頼区間を算出した。

結果 : 2009 年度の粗有病率（/10 万人/年）と粗罹患率（/10 万人/年）は、それぞれ 9.9（9.7–10.1）と 2.2（2.1–2.3）であった。年齢別にみると、粗有病率および粗罹患率ともに、70 歳代で最も高く、性別をみると、男性の方が女性より約 1.5 倍ほど高かった。47 都道府県別に標準化罹患比をみてみると、男性では沖縄、奈良、和歌山、新潟、女性では熊本で高かった。人種間の比較を行うために、米国の 2000 年基準人口を用いて標準化罹患率（/10 万人/年）を求めたところ、2.3（2.2–2.4）という結果であった。

結論 : 本研究は、日本人における年間有病率および罹患率に関する、初めての報告と言える。国内においては、一部の都道府県に高い罹患率が見られ、ALS の罹患に地域差が示唆された。海外との比較においては、欧米の白色人種に比べると、その罹患率は極めて低いことが示唆された。

キーワード

ALS、筋萎縮性側索硬化症、疫学、罹患率、日本
